# Human Germline Interventions–Think First

**DOI:** 10.3389/fgene.2016.00081

**Published:** 2016-05-09

**Authors:** Elisabeth Hildt

**Affiliations:** Center for the Study of Ethics in the Professions, Illinois Institute of TechnologyChicago, IL, USA

**Keywords:** germline, gene editing, CRISPR, gene therapy, human embryo, ethics, societal implications, ELSI

Recent studies using gene editing tools such as CRISPR/Cas9 have shown an enormously broad spectrum of possible future applications. This genetic engineering technique allows researchers to easily disable, insert, or replace DNA fragments, while raising risks such as incomplete or inaccurate editing, off-target mutations, and other unintended consequences. Whereas there is consensus that possible future uses in somatic cells hold enormous promise in clinical contexts, human germline interventions, i.e., the genetic modification of human gametes or embryos, are highly controversial (Baltimore et al., 2015; Lander, 2015; Lanphier et al., 2015; Miller, 2015; Pollack, 2015; Savulescu et al., 2015).

Up to now, at least in Western countries, there has been a broad consensus to ban interventions that aim to modify the human germline. For example, the Council of Europe's Convention on Human Rights and Biomedicine from 1997 (http://conventions. coe.int/Treaty/en/Treaties/Html/164.htm) reads in Article 13:

“An intervention seeking to modify the human genome may only be undertaken for preventive, diagnostic, or therapeutic purposes and only if its aim is not to introduce any modification in the genome of any descendants.”

In December 2015, the Council of Europe's Committee on Bioethics adopted a Statement on Genome Editing Technologies (https://rm.coe.int/CoERMPublicCommonSearchServices/DisplayDCTMContent?documentId=090000168049034a) that stresses the relevance of the Convention on Human Rights and Biomedicine with regard to gene editing techniques.

Article 1 of UNESCO's Universal Declaration on the Human Genome and Human Rights from 1997 states (http://portal.unesco.org/en/ev.php-URL_ID=13177&URL_DO=DO_TOPIC&URL_SECTION=201.html):

“The human genome underlies the fundamental unity of all members of the human family, as well as the recognition of their inherent dignity and diversity. In a symbolic sense, it is the heritage of humanity.”

This can be seen in the context of UNESCO's Universal Declaration on Bioethics and Human Rights (2005) (http://portal.unesco.org/en/ev.php-URL_ID=31058&URL_DO=DO_TOPIC&URL_SECTION=201.html) which says in Article 16: “The impact of life sciences on future generations, including on their genetic constitution, should be given due regard.”

In October 2015, a UNESCO panel of experts called for a temporary ban on editing the human germline (http://www.unesco.org/new/en/social-and-human-sciences/themes/bioethics/sv0/news/unesco_panel_of_experts_calls_for_ban_on_editing_of_human_dna_to_avoid_unethical_tampering_with_hereditary_traits/#.VwgJUvkrJaQ).

Many countries ban germline genetic modification either by law or by guidelines, while other countries are ambiguous about the legal status of germline modifications (cf. Araki and Ishii, 2014).

These regulations and agreements are to be seen against the background of a long interdisciplinary debate on ethical and societal issues of germline interventions that began in the 1980s. In this debate, a broad spectrum of topics has been discussed, including medical issues concerning safety and efficacy, possible health benefits, risks and unintended consequences for future generations, and ethical issues such as individual and collective responsibility, reproductive autonomy, human dignity, the moral status of human embryos, human nature, tampering with human evolution, playing God, eugenics, discrimination, human enhancement, and transhumanist visions.

Up to a few months ago, in the absence of a technique to modify the human germline by disabling, inserting, or replacing DNA fragments, the debate on human germline interventions seemed to be rather academic and theoretical, far away from any reality. It is the advent of this new technique of gene editing and the observation that it can in principle be used to modify the DNA of the resulting organism and its offspring that revitalizes the debate on germline interventions and gives it a new direction. In this, the mere availability of a technique that allows us to modify the human germline seems to provide a strong push toward human germline interventions. Whereas the plausibility or implausibility of the various arguments in the debate around human germline interventions has not changed, the situation around this debate is quickly evolving: the worldwide scientific community watches an exciting scientific breakthrough, scientists are engaging in a new field of research, and manuscripts are appearing that put pressure on scientific journals to publish them (Sharma and Scott, 2015). All of this seems to develop a strong momentum of its own, resulting in a rush toward using this new technique for germline intervention.

Recently, a group of Chinese researchers published the results of human germline modification experiments involving tripronuclear zygotes, i.e., zygotes that are not capable of developing normally (Liang et al., 2015). In December 2015, the members of the Organizing Committee for the International Summit on Human Gene Editing, in which leading researchers in the field participated, published a summit statement (http://www8.nationalacademies.org/onpinews/newsitem.aspx?RecordID=12032015a). In this, they do not exclude *in-vitro* research involving human germline interventions as long as the modified cells are not used to establish a pregnancy, but for the time being argue against any clinical use of germline editing. During the past few months, also others have spoken in favor of running *in-vitro* human germline genetic interventions (Baltimore et al., 2015; Miller, 2015). Some scientists have argued toward a general ban of human germline modifications using the CRISPR/Cas9 technique and similar techniques, however, (Lanphier et al., 2015; Pollack, 2015).

There is an urgent need to step back and think about what is going on. The recent move toward running *in-vitro* human germline interventions is a remarkable development. This is particularly true as currently, in view of the high risks and unresolved practical, societal and ethical issues involved, nobody would ever argue toward attempting human germline modifications that involve initiating a pregnancy. However, the move toward *in-vitro* human germline interventions attempts to set the stage for future uses involving human reproduction. Even if *in-vitro* studies involving only early stages of human embryonic development are far less problematic from a practical and ethical point of view than research involving the initiation of a pregnancy or the birth of a child, they are still highly problematic. *In-vitro* research with early human embryos clearly is not done in a vacuum but has to be seen in the broader context of the attempt to find possible future applications involving human reproduction.

## There are no new powerful arguments in favor of human germline interventions

Are there any new powerful arguments in favor of human germline interventions? I do not see any.

The main argument put forward in favor of clinical germline interventions is that they may serve to prevent diseases and avoid possible suffering in future generations. It has been proposed that germline interventions might be an appropriate tool for the prevention of monogenic diseases in situations in which there is a 100% probability that the disease will be passed on to the progeny. This is the case when one parent is homozygous for a dominant disease or both parents are homozygous for an autosomal recessive disease. Sickle cell anemia or recessive deafness would be examples of the latter (Wivel and Walters, 1993; Lander, 2015; Miller, 2015). However, situations like these are very rare. It is questionable whether there would be broader justifiable medical uses for germline interventions, especially in view of the availability of genetic testing and pre-implantation genetic diagnosis.

Furthermore, speculations on possible future additional applications seem to play a considerable role in the rush toward human germline interventions. These include the possibility of risk reduction in widespread multigenic and multifactorial disorders such as cardiovascular disease or cancer. However, interactions between the numerous genetic and environmental factors are most often very complicated so that it is questionable whether the risks involved would ever outweigh the potential benefits (Wivel and Walters, 1993; Lander, 2015).

In addition, germline interventions may be considered a future option for parents who attempt to specify more than one trait of their future offspring. It might be easier to genetically modify the traits in question than to attempt to select embryos with the desired traits from a limited number of available embryos by using pre-implantation genetic diagnosis (cf. Savulescu et al., 2015).

Beyond that, according to further speculations, germline gene editing may render it possible to influence a broad spectrum of traits or enhance human capabilities. With germline genetic interventions, the choices available for future parents would increase considerably. This opens up the debate for speculations on improving mankind, genetic enhancements, eugenics and designer babies, all of these clearly underlining the manifold ethical and societal issues involved in human germline interventions. Interestingly, with regard to human germline interventions, several authors consider a development toward genetic enhancements to be inevitable (Wivel and Walters, 1993; Baylis and Robert, 2004).

## Avoid premature experiments

In his article “Human gene therapy: scientific and ethical considerations” published in 1985, W. French Anderson named three conditions that should be met prior to any attempt to undergo germline gene therapy in humans: Substantial previous experience with somatic cell gene therapy in humans proving safety and efficacy of the approach; adequate animal research that shows the reproducibility, reliability, and safety of germline therapeutic interventions; and the informed public approval of the procedure (Anderson, 1985).

In spite of the fact that neither comprehensive knowledge with gene editing of somatic cells nor with germline applications in animal studies is available at the moment, *in-vitro* experiments involving human germline modifications have already been initiated (Liang et al., 2015)^1^. Whereas *in-vitro* studies are far less problematic than studies using gene editing in reproductive contexts, it should not be forgotten that *in-vitro* studies involving human embryos bear considerable practical and ethical issues when it comes to the creation and destruction of human embryos for research purposes or to the use of surplus *in-vitro* fertilization (IVF) embryos. For the time being, considering the moral status of human embryos and the lack of adequate experience with gene editing tools, *in-vitro* human germline interventions involving human embryos can hardly be justified.

Furthermore, before overriding general standards that have been agreed upon widely in Western societies, it will be absolutely necessary to thoroughly reflect on the issues involved. For this, a broad societal debate on the practical, societal, and ethical issues in human germline interventions involving the various groups within society is urgently needed. Beyond an interdisciplinary academic debate involving disciplines such as medicine, natural sciences, humanities, social sciences and the law, it will be particularly important for scientists to inform the public of the ongoing development and to engage in a serious dialog with critical stakeholders.

Only widely held and well informed public approval can legitimize researchers to go on in a sensitive field like this which has the power to affect society as a whole. For the time being, however, not much is known on the opinion of the public. Up to now, the controversy on gene editing and human germline interventions is dominated by scientists involved in the field. The general public, it seems, has difficulties keeping abreast with the quick development and understanding the issues at stake.

For sure, a balanced debate that involves a broad spectrum of the various societal groups will have an open outcome. It may be the case that societal values change over time, that the balancing of pros and cons is done differently now than it was done in the past, or that new and important arguments in favor of or against germline interventions will come up. But all of this needs careful and thorough reflection.

In view of the unresolved practical, ethical and societal issues, for the time being, it is more than advisable to refrain from any experiments involving human germline interventions. Instead: Think first. Don't hastily override existing standards broadly agreed upon.

## Author contributions

The author confirms being the sole contributor of this work and approved it for publication.

### Conflict of interest statement

The author declares that the research was conducted in the absence of any commercial or financial relationships that could be construed as a potential conflict of interest.

## Footnotes

^1^Albeit in this particular study, the tripronuclear zygotes involved were not capable to develop normally.
